# Changes of antioxidant enzymes in the kidney after cardiac arrest in the rat model

**DOI:** 10.1590/1414-431X2023e12408

**Published:** 2023-02-10

**Authors:** J.H. Lee, M.S. Islam, Y.J. Yoo, S.E. Kim, R.H. Kim, Y.J. Jang, S.H. Lee, H.P. Hwang, H.Y. Shin, J.H. Hwang, K. Kim, B.Y. Park, D. Ahn, Y. Lee, T. Kim, I.S. Kim, J.C. Yoon, H.J. Tae

**Affiliations:** 1 Department of Anesthesiology and Pain Medicine, Research Institute of Clinical Medicine, Jeonbuk National University, Biomedical Research Institute, Jeonbuk National University Hospital, Jeonju, Korea; 2 Department of Veterinary Medicine and Institute of Animal Transplantation, Jeonbuk National University, Iksan, Jeollabuk-do, Korea; 3 Department of Emergency Medicine, Research Institute of Clinical Medicine, Jeonbuk National University and Biomedical Research Institute, Jeonbuk National University Hospital, Jeonju, Korea; 4 Department of Surgery, Jeonbuk National University Medical School and Hospital, Jeonju, Korea; 5 Animal Model Research Group, Jeonbuk Branch Institute, Korea Institute of Toxicology, Jeongup, Jeonbuk, Korea; 6 Department of Thoracic and Cardiovascular Surgery, Research Institute of Clinical Medicine, Jeonbuk National University-Biomedical Research Institute, Jeonbuk National University Hospital, Jeonju, Korea

**Keywords:** Cardiac arrest, Ischemia-reperfusion, Oxidative stress, Antioxidant enzymes, Renal injury

## Abstract

Globally, cardiac arrest (CA) is a leading cause of death and disability. Asphyxial CA (ACA)-induced kidney damage is a crucial factor in reducing the survival rate. The purpose of this study was to investigate the role of antioxidant enzymes in histopathological renal damage in an ACA rat model at different time points. A total of 88 rats were divided into five groups and exposed to ACA except for the sham group. To evaluate glomerular function and oxidative stress, serum levels of blood urea nitrogen (BUN) and creatinine (Crtn) and malondialdehyde (MDA) levels in renal tissues were measured. To determine histopathological damage, hematoxylin and eosin staining, periodic acid-Schiff staining, and Masson's trichrome staining were performed. Expression levels of antioxidant enzymes including superoxide dismutase-1 (SOD-1), superoxide dismutase-2 (SOD-2), catalase (CAT), and glutathione peroxidase (GPx) were measured by immunohistochemistry (IHC). Survival rate of the experimental rats was reduced to 80% at 6 h, 55% at 12 h, 42.9% at 1 day, and 33% at 2 days after return of spontaneous circulation. Levels of BUN, Crtn, and MDA started to increase significantly in the early period of CA induction. Renal histopathological damage increased markedly from 6 h until two days post-CA. Additionally, expression levels of antioxidant enzymes were significantly decreased at 6 h, 12 h, 1 day, and 2 days after CA. CA-induced oxidative stress and decreased levels of antioxidant enzymes (SOD-1, SOD-2, CAT, GPx) from 6 h to two days could be possible mediators of severe renal tissue damage and increased mortality rate.

## Introduction

Ischemia and reperfusion (I/R)-mediated damage has been identified as one of the major causes of debilitating disease and death in several pathological conditions, including myocardial infarction, ischemic stroke, and cardiac arrest (CA) (1). An ischemic environment caused by an interruption in the blood flow induces tissue injury initially with subsequent damage prompted by reperfusion (2). I/R injury is a multifocal process involving numerous cell types and signaling pathways (3). Among them, oxidative stress-mediated injury after I/R has been well established (1). Since I/R is an enormously complex process, its pathophysiology is not yet fully understood.

After cardiac arrest, the mortality rate increases in those patients who immediately achieve the return of spontaneous circulation (ROSC), which seems to be a possible trigger that involves multiple organs. Although sustained ischemic conditions in the whole body primarily mediate global tissue and organ injury, further damage can occur during and after reperfusion (4,5). Previous studies have found post-cardiac arrest syndromes (PCAS) in various organs, especially in the heart, brain, and renal tissue (6,7). However, most studies were focused on brain injury. The occurrence and effect of I/R injury in the kidney after CA are not described well (8).

I/R is mediated by oxidative stress, which may cause injury to insulted tissues. The damage caused by the return of oxygen to an ischemic tissue is far greater than the injury caused by ischemia alone (9). However, in a two-phase pattern, oxidative stress can lead to I/R injury-provoked damage. Oxidative stress directly induces cytotoxicity by producing free radicals and indirectly triggers post-ischemia-reperfusion inflammatory injury by recruiting inflammatory mediators through redox-mediated signaling pathways (9,10). These oxidative stress and inflammatory reactions have been associated with the development of multiple organ failure (11).

Reactive oxygen species (ROS) generated by sudden recirculation of oxygen have been found to play a significant role in the pathophysiology of I/R injury (12). Moreover, excess production of ROS can stimulate lipid peroxidation, DNA damage, apoptosis, and necrosis and trigger cellular death in several ways (13,14). To protect ROS-mediated injury, it is important to neutralize the effects of ROS. Antioxidant enzymes such as superoxide dismutases (SODs), catalase (CAT), and glutathione peroxidase (GPx) can provide a front line of defense against ROS-mediated injury (15,16). SODs, CAT, and GPx are a group of metalloenzymes that catalyze the dismutation of superoxide anion (-O_2_) into hydrogen peroxide (H_2_O_2_) and subsequently increase the breakdown of H_2_O_2_ to water and molecular oxygen (O_2_) (16-18).

However, in a previous experiment, antioxidant enzymes and their relationships with renal injury were observed only one day after induction of asphyxial CA enzymes (19). A time-course study was not performed. Thus, the aim of this study was to examine the changing pattern of antioxidant enzymes (SOD-1, SOD-2, CAT, Gpx) in rat kidneys following asphyxial CA in a time-dependent manner.

## Material and Methods

### Experimental animals and groups

All experimental methods of this investigation were approved by Jeonbuk National University (approval No. CBNU 2020-084) based on guidelines of ethics and scientific care provided by the Institutional Animal Care and Use Committee (IACUC) at Jeonbuk National University. The experimental Sprague Dawley male rats (body weight: 270-330 g) were 7 weeks old. They were provided by the Experimental Animal Center of Jeonbuk National University (Iksan campus, South Korea). A total of 88 rats were used in this study and assigned to five groups. Eight rats were used for the sham group and the remaining 80 rats were used for CA surgery. CA-operated rats were sacrificed at 6 h (n=8), 12 h (n=8), 1 day (n=8), and 2 days (n=8) following ROSC.

### Induction of CA and cardiopulmonary resuscitation (CPR)

**Figure 1:**
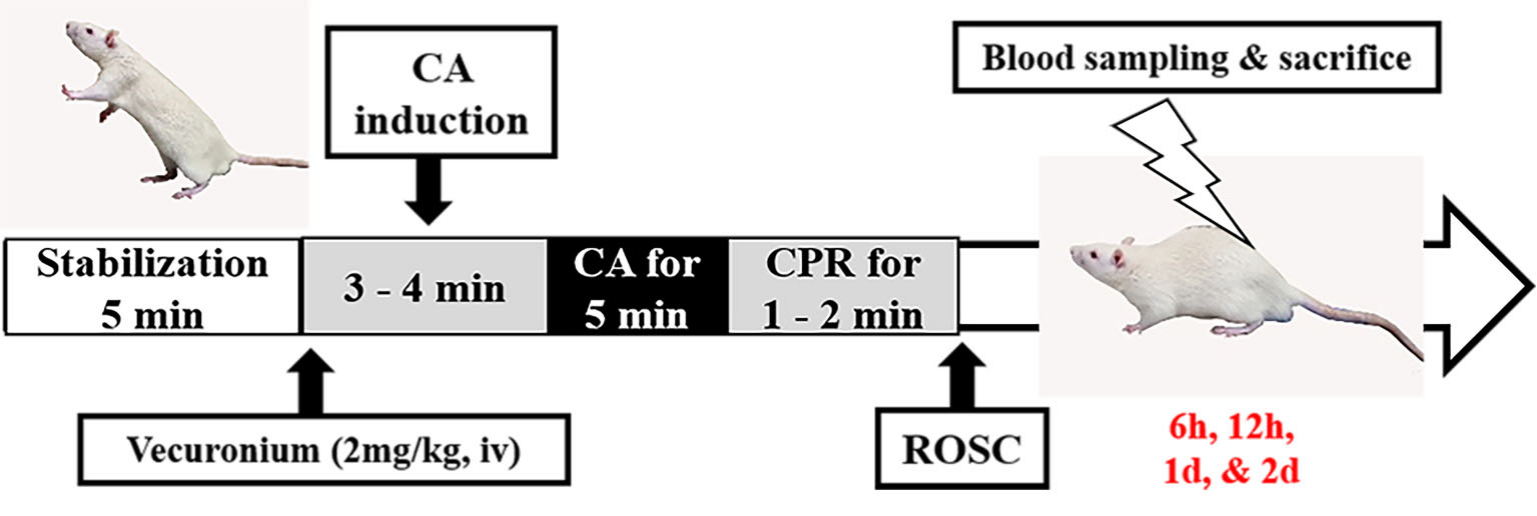
Schematic diagram of the cardiac arrest (CA) model in rats with the pragmatic time used for animal stabilization, CA induction, cardiopulmonary resuscitation (CPR), rate of spontaneous circulation (ROSC), and sacrifice.

CA induction and CPR were performed following an established protocol (20). Anesthesia was maintained using a rodent ventilator (Harvard Apparatus, USA). Heating pads were used to maintain body temperature (37±0.5°C). During the experimental period, peripheral oxygen saturation (SpO_2_) and electrocardiogram (ECG) data were recorded frequently. The right femoral vein was exposed to insert the cannula for intravenous injection. Mean arterial pressure (MAP) was measured by cannulation of the left femoral artery. To induce CA in rats, intravenous vecuronium bromide (2 mg/kg, Gensia, Sicor Pharmaceuticals, USA) was inserted, and mechanical ventilation was stopped (19). CA was confirmed by MAP values lower than 25 mmHg after 3-4 min of a stabilization period. Five minutes after induction of CA, epinephrine (0.005 mg/kg, Sigma, USA) and sodium bicarbonate (1 mEq/kg, Sigma) were injected intravenously (19). Mechanical chest compression (Jeung Do Bio & Plant Co., Ltd., Korea) was done at 300/min with 100% oxygen supply. When rats became thermodynamically stable, they were sacrificed at specific time points (Figure 1).

### Evaluation of serum levels of urea nitrogen and creatinine

For euthanasia, 30% urethane (Sigma) was used. Then 3-5 mL of blood was collected from the inferior vena cava. Serum was separated from blood by centrifugation at 3400 *g* or 15 min and used to determine blood urea nitrogen (BUN) and creatinine (Crtn) levels with an Automatic Analyzer 7020 (Hitachi, Japan).

### Evaluation of malondialdehyde (MDA)

Levels of MDA as an oxidative stress marker in renal tissues were evaluated following instructions of a commercial kit (Cayman Chemical, USA). In brief, renal tissues were homogenized and centrifuged at 8832 *g* for 10 min. The supernatant was collected and kept at -80°C for experimental analysis. Using a tunable versus max microplate reader (Cayman Chemical), the absorbance of MDA was measured at 535 nm.

### Hematoxylin and eosin (H&E), periodic acid-Schiff (PAS), and Masson's trichrome staining

Both kidneys were removed carefully after sacrificing rats without any damage and fixed with 10% neutral buffered formalin. Paraffin-embedded blocks were then made and sectioned (5 µm in thickness) for H&E, PAS, and Masson's trichrome staining to examine histopathological changes, glomerular basement membrane, and interstitial fibrosis, respectively (21). Images of stained kidney sections were taken using a Leica DM 2500 microscope (Leica Microsystems, Germany). For H&E-stained sections, ×400 magnification was used. For PAS- and Masson's trichrome-stained sections, ×1,000 magnifications were used. For each rat, separate slides were prepared. Ten different fields of the same slide were then examined. Two experienced renal pathologists assessed histopathological changes via quantitative tubulointerstitial injury measurement by counting numbers of apoptotic and necrotic cells, determining loss of tubular brush border, tubular dilatation, cast formation, and neutrophil infiltration, and examining glomeruli basement membrane thickness in a double-blinded fashion. The scoring was done based on level of damage: 0=none; 1=0-10%; 2=11-25%; 3=26-45%; 4=46-75%; and 5=76-100% (22).

### Immunohistochemistry

To observe the expression pattern of antioxidant enzymes in renal tissues, immunohistochemistry was performed according to our published protocol (23). In short, xylene and ethanol were used for deparaffinization and dehydration of tissue sections, respectively. Antigen retrieval and quenching were done using citrate buffer and 3% hydrogen peroxide, respectively. Tissue sections were blocked with goat serum and then incubated with primary antibodies for SOD-1 (Rabbit, Abcam, USA, #cat ab13498), SOD-2 (Rabbit, Abcam, #cat ab13533), CAT (Rabbit, Abcam, #cat ab16731), and Gpx (Rabbit, Abcam, #cat ab22604) overnight at 4°C according to the dilution recommended by the company. Afterward, tissue sections were incubated with a secondary antibody (Vector Laboratories Inc., USA) and vectastain ABC reagent (Vector Laboratories Inc.) at room temperature for 1 h. For the brown staining of immunoreactive tissue sections, diaminobenzidine (DAB, Sigma-Aldrich) was used. Counterstain was done using hematoxylin. Finally, sections were mounted onto glass slides after dehydration and cleaned with ethanol and xylene bath. A Leica DM 2500 microscope (Leica Microsystems) was used to capture images of immunoreactive tissue sections. ImageJ threshold analysis software (ij152-win-Java8; NIH, USA) was used to analyze the relative absorbance in percentage.

### Statistical analysis

GraphPad Prism 5.0 (USA) was used to analyze data. Data are reported as means±SE. Survival rates were measured using Kaplan-Meier statistics and log-rank tests. Comparison of data among groups was performed using one-way analysis of variance (ANOVA) followed by Bonferroni's multiple comparison tests. For all analyses, statistical significance was considered when the P-value was less than 0.05.

## Results

### Physiological variables

There were non-significant (P>0.05) differences in baseline characteristics between CA-operated groups and the sham group (Table 1). MAP and SpO_2_ with isoelectric ECG were used to confirm CA. Changes were observed for ECG, MAP, and SpO_2_ as expected according to the protocol. The survival rate of rats was 80% at 6 h, 55% at 12 h, 42.9% at 1 day, and 33% at 2 days after ROSC. At baseline and after ROSC, body temperature, body weight, and heart rate did not change significantly. The room temperature was kept stable during the experiment.

**Table 1:** Physiological condition before (sham) induction of cardiac arrest and after in rats (n=8).

Parameter	Sham	6 h	12 h	1 d	2 d
Body weight (g)	328.4±17.3	330.3±16.7	327.9±13.2	331.2±17.0	330.4±12.1
SpO_2_	97.2±0.7	97.8±0.6	96.4±0.2	96.6±1.7	98.0±1.2
MAP (mmHg)	122.2±0.5	119.8±1.4	116.2±1.3	120.1±1.4	120.6±0.5
Asphyxia time to CA (s)	-	157.3±13.3	158.0±14.3	162.0±16.3	153.8±8.79
CPR time (s)	-	75.2±10.6	74.4±10.6	71.1±12.5	73.4±11.0
Survival rate (%)	100.0	80.0	55.0	42.9	33.0

Data are reported as means±SE. There were no significant differences between groups. SpO_2_: oxygen saturation; MAP: mean arterial pressure; CA: cardiac arrest; CPR: cardiopulmonary resuscitation; d: day.

### Renal function evaluation and MDA levels in renal tissues

**Figure 2:**
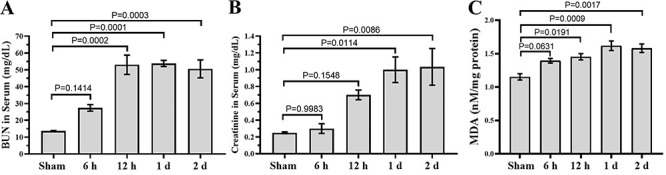
Effects of return of spontaneous circulation (ROSC) after induction of cardiac arrest (CA) on blood urea nitrogen (BUN), creatinine, and malondialdehyde (MDA) levels on blood serum. Data are reported as means±SE (n=8). P<0.05, ANOVA followed by Bonferroni's multiple comparison tests.

Serum levels of BUN and Crtn were significantly (P<0.05) increased at 6 h, 12 h, 1 day, and 2 days after ROSC. The peak level of BUN was at 12 h after ROSC, and it was maintained for up to 2 days. The peak level of Crtn was at 1 day after ROSC, and it was maintained for up to 2 days (Figure 2A and B). MDA concentrations at 6 h, 12 h, 1 day, and 2 days after ROSC were significantly (P<0.05) increased in kidneys of rats with CA-induced ischemia compared with those in the sham group (Figure 2C).

### Histopathological findings

**Figure 3:**
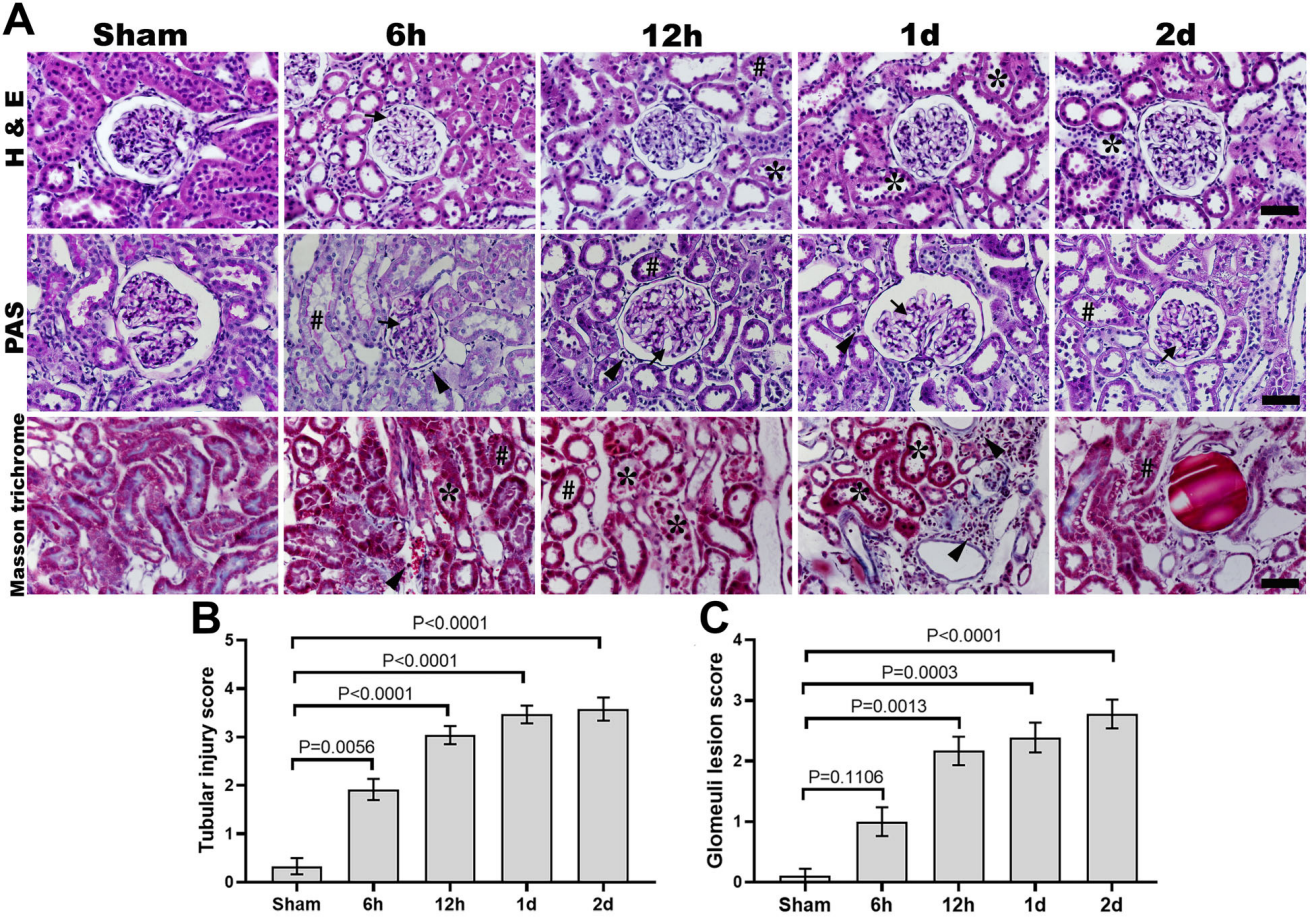
Effects of return of spontaneous circulation (ROSC) after induction of cardiac arrest (CA) on renal histopathological changes. H&E, PAS, and Masson trichrome staining (**A**) showed significantly augmented tubular injury and damage in the glomerulus in the CA-operated groups (6 h, 12 h, 1 day, 2 days) compared to the sham group. Graphs represent the tubular injury score (**B**) and glomerular basement membrane damage score (**C**). Original magnification: ×400. Scale bar: 50 µm. Data are reported as means±SE (n=8). P<0.05, ANOVA followed by Bonferroni's multiple comparison tests. (★) Indicates loss of brush border with necrosis, (#) indicates irregular brush borders, (→) indicates dilated glomerular capillaries, and (▸) indicates infiltration of inflammatory cells in CA-operated groups.

Histopathology results showed that brush borders of epithelial cells of renal tubules were completely lost with dilatation and infiltration of inflammatory cells in glomerular capillaries and acute tubular necrosis following ROSC. H&E score showed significant damage to proximal and distal convoluted tubules of the renal cortex at 6 h, 12 h, and 2 days post-CA (Figure 3). PAS staining score showed that the diameter of glomeruli capillaries and the thickness of the glomerular basement membrane were markedly increased at 6 h, 12 h, 1 day, and 2 days after ROSC compared with the sham. Masson's trichrome staining revealed increased interstitial fibrosis in the renal cortex following ROSC in a time-dependent manner (Figure 3).

### Immunohistochemical analysis of antioxidant enzymes

**Figure 4:**
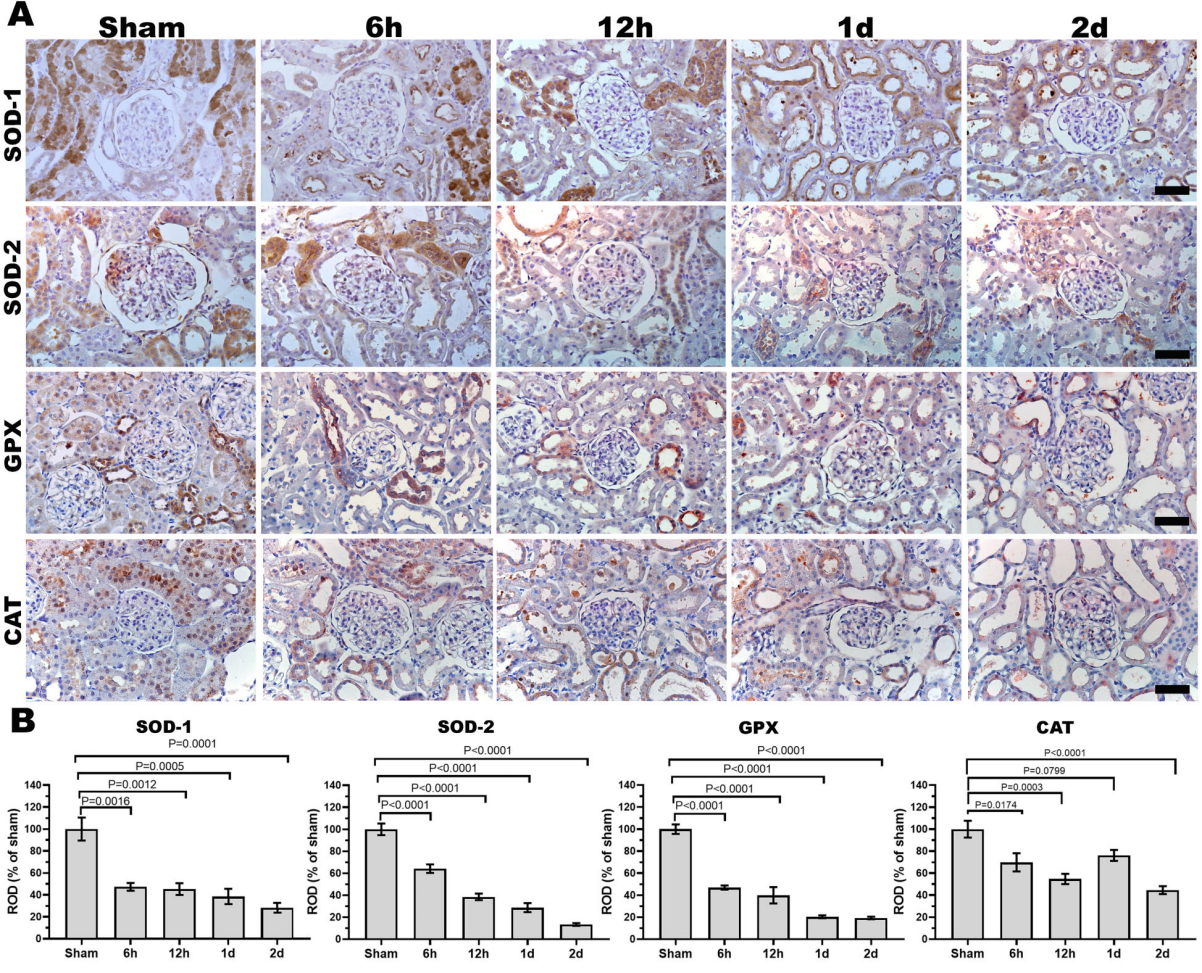
Effects of return of spontaneous circulation (ROSC) after induction of cardiac arrest (CA) on renal antioxidant enzymes. **A**, Immunohistochemistry showing significantly decreased expression of antioxidant enzymes superoxide dismutase (SOD)-1, SOD-2, glutathione peroxidase (GPx), and catalase (CAT) in the CA-operated groups (6 h, 12 h, 1 day, 2 days) compared to the sham group. Graphs represent the relative optical density (ROD%) of (**B**) SOD-1, SOD-2, GPx, and CAT expression. Original magnification: ×400. Scale bar: 50 µm. Data are reported as means±SE (n=8). P<0.05, ANOVA followed by Bonferroni's multiple comparison tests.

Immunoreactive antioxidant enzymes SOD-1, SOD-2, CAT, and GPx were significantly reduced in renal tissues of CA-operated groups. In the sham group, expression levels of antioxidant enzymes were markedly higher in tubular cells. However, other groups displayed lower numbers of tubular cells stained with SOD-1, SOD-2, CAT, and GPx in a time-dependent manner. Immunoreactive SOD-1 and SOD-2 expression levels were significantly decreased at 6 h and maintained for 2 days after ROSC. GPx and CAT expression levels started to decrease at 6 and 12 h and continued for 2 days (Figure 4).

## Discussion

The survival rate of out-of-hospital CA patients who have received CPR from ambulance personnel and surviving admission ranges from 14 to 39%, with more than half of them dying within the first 24 h after arriving at the hospital (24). The survival rate in CA animal models varies according to the methodology or animals employed. In the present study, the survival rate declined in a time-dependent manner, reaching 33% at two days post-CA. Previous studies have also shown the same pattern of survivability (25,26). Therefore, our asphyxial CA model was suitable for studying CA patients.

Severe damage is found mostly in the heart and brain following I/R injury after CA (27). Nonetheless, some research has found that acute renal impairment affects neurological recovery (28). Furthermore, acute renal damage is increased in 43% of patients resuscitated after CA, with more than 75% of such damage occurring within three days (29). Thus, it is important to focus on acute renal damage following CA and CPR. In the present study, we found severe histological changes in kidney tissues. Jawad et al. (26) and Kim et al. (19) have reported the same pattern of histopathological changes in renal tissues after induction of asphyxial CA. Creatinine and BUN are the most frequently utilized endogenous indicators for assessing glomerular function (30). These are markedly augmented after ROSC in CA hospital patients and in an induced-CA rat model (26). Previous studies have also reported that BUN and creatine levels are increased at 1 day and 2 days after ROSC in an asphaxial CA rat model (31,32). Our experimental data were also similar to studies using a traditional I/R model. There were only differences in histopathological damage in renal tissues. BUN and Crtn levels started to increase in an earlier period of 6 h after ROSC. Results of BUN and Crtn levels and histological analysis confirmed that acute kidney injury could progress in the initial period of CA and continue for 2 days in asphyxial CA.

The involvement of oxidative stress is crucial in the development of renal ischemic injury, which is pathologically prompted by excess generation of ROS and reactive nitrogen species (RNS) (33). Those superoxides are extremely bioactive oxygen molecules and vastly linked to renal tissue damage (34). Overproduction of ROS ultimately induces DNA degradation, protein inactivation, and structural and functional disruption of renal tubular cells by widespread membrane lipid peroxidation (35,36). Increased ROS can also lead to MDA generation and severely weaken the antioxidant enzyme system (37). In the present study, MDA was markedly increased in CA groups compared with the sham group, resembling results of a previous study (26). Thus, asphyxial CA can increase oxidative stress in a time-dependent manner and provoke renal damage.

Detrimental consequences of oxidative stress can be reduced by enzymatic antioxidant systems known to scavenge ROS. Antioxidant enzymes such as SODs, CAT, and GPx can inhibit ROS-mediated injury. SODs are the most dominant antioxidant enzymes in cells. They can initiate neutralization of ROS-induced toxicity and accelerate the neutralization and dismutation of superoxide anion (-O_2_), which is highly detrimental, into hydrogen peroxide (H_2_O_2_) and molecular oxygen (O_2_) (16). To reduce toxic effects of H_2_O_2_ on tissues, CAT can increase the breakdown of H_2_O_2_ into water and molecular oxygen, which completes the detoxification process similar to SOD (17). However, in mammals, CAT is restricted to peroxisomes, making it improbable to interact with SOD-produced hydrogen peroxide (38). CAT might decompose peroxynitrite and oxidize nitric oxide to nitrite to balance oxidation of NO (39). In addition, GPx is a vital intracellular antioxidant enzyme that can also intensify the degradation of H_2_O_2_ to H_2_O and lipid peroxides to their equivalent alcohols, mainly in the mitochondria (18). The present experiment revealed that antioxidant defense systems were altered by asphyxial CA-mediated I/R injury. The change in the potentiality of antioxidant enzymes suggested the role of ROS in the pathogenesis of asphyxial CA-mediated I/R renal injury. Our study revealed that the immunoreactivity of antioxidant enzymes including SOD-1, SOD-2, CAT, and GPx started to decline at an early stage of CA (6 h) and continued to decline significantly up to 2 days after ROSC compared with the sham group. Expression patterns of antioxidant enzymes in the asphyxial CA model are similar to those in the ischemic acute kidney injury model. A previous experiment has found that antioxidant enzymes including SOD-1, SOD-2, CAT, and GPx in kidneys exposed to ischemia for 30, 60, 90 min, 2 h, and 24 h are reduced significantly (40). Kim et al. (19) have shown that immunoreactivity of antioxidant enzymes (SOD-1, SOD-2, CAT, GPx) in renal tissues are decreased at 1 day after I/R induction by asphyxial CA and that hypothermia treatment can increase the expression of antioxidants enzymes.

## Conclusion

CA-induced ischemia significantly increased oxidative stress in renal tissues during the early period (6 h). Oxidative stress-mediated markers could be involved in the reduction of activities of antioxidant enzymes such as SOD-1, SOD-2, GPx, and CAT, ultimately increasing renal injury in a time-dependent manner. They could be potential factors for a low survival rate. Further studies are needed to explore the exact mechanism involved.
